# Genetic and Physiological Characterization of the Pentose Phosphate Pathway in the Yeast *Kluyveromyces lactis*

**DOI:** 10.3390/ijms26030938

**Published:** 2025-01-23

**Authors:** Laura-Katharina Bertels, Stefan Walter, Jürgen J. Heinisch

**Affiliations:** 1 Department of Genetics, Faculty of Biology and Chemistry, University of Osnabrück; Barbarastr. 11, D-49076 Osnabrueck, Germany; lbertels@uni-osnabrueck.de; 2 Facility for Mass Spectrometry, Faculty of Biology and Chemistry, University of Osnabrück; Barbarastr. 11, D-49076 Osnabrueck, Germany; stwalter@uni-osnabrueck.de

**Keywords:** pentose phosphate pathway, essential genes, *tetOFF* system, proteome, ß-oxidation

## Abstract

The pentose phosphate pathway (PPP) is essential for human health and provides, amongst others, the reduction power to cope with oxidative stress. In contrast to the model baker’s yeast, the PPP also contributes to a large extent to glucose metabolism in the milk yeast *Kluyveromyces lactis*. Yet, the physiological consequences of mutations in genes encoding PPP enzymes in *K. lactis* have been addressed for only a few. We here embarked on a systematic study of such mutants, deleting *ZWF1*, *SOL4*, *GND1*, *RKI1*, *RPE1*, *TKL1*, *TAL1*, and *SHB17*. Interestingly, *GND1*, *RKI1*, and *TKL1* were found to be essential under standard growth conditions. Epistasis analyses revealed that a lack of Zwf1 rescued the lethality of the *gnd1* deletion, indicating that it is caused by the accumulation of 6-phosphogluconate. Moreover, the slow growth of a *tal1* null mutant, which lacks fructose-1,6-bisphosphate aldolase, was aggravated by deleting the *SHB17* gene encoding sedoheptulose-1,7-bisphosphatase. A mitotically stable *tetOFF* system was established for conditional expression of *TAL1* and *TKL1*, encoding transaldolase and transketolase in the non-oxidative part of the PPP, and employed in a global proteome analysis upon depletion of the enzymes. Results indicate that fatty acid degradation is upregulated, providing an alternative energy source. In addition, *tal1* and *tkl1* null mutants were complemented by heterologous expression of the respective genes from baker’s yeast and humans. These data demonstrate the importance of the PPP for basic sugar metabolism and oxidative stress response in *K. lactis* and the potential of this yeast as a model for the study of PPP enzymes from heterologous sources, including human patients.

## 1. Introduction

Yeasts are unicellular eukaryotes comprising at least 1500 species, which divide by budding, belong to the fungal kingdom, inhabit vastly different environments, and dispose of a wide range of physiological activities [1]. The best studied member, if not the best understood eukaryote, is undoubtedly the wine, beer, and baker’s yeast *Saccharomyces cerevisiae*. Its “scientific career” was historically seeded by the long-term worldwide application in the production of bread and beverages, which itself is based on the high capacity for sugar degradation through glycolysis and alcoholic fermentation. In fact, *S. cerevisiae* employs this route at high sugar concentrations independent from oxygen availability. This phenomenon is known as the Crabtree effect, and stands in contrast to the Pasteur effect, which designates the preferential energy production by respiration in the presence of oxygen. Ironically, the latter is not shown by the very yeast Pasteur employed for his studies [2].

**Figure 1:**
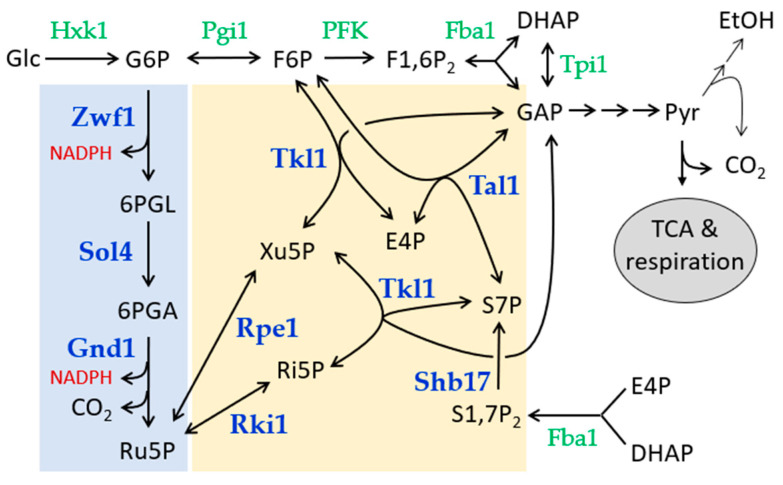
Routes of glucose catabolism in *Kluyveromyces lactis*. Simplified overview of the enzymes involved in the initial steps of glycolysis (green letters) and those of the pentose phosphate pathway (PPP) investigated in this work (bold blue letters). The oxidative part of the PPP is shaded in pale blue, and the non-oxidative part in pale yellow. Arrows between metabolites indicate their interconversion, with double arrows designating reversible, and pointed arrows the irreversible reactions. Abbreviations of enzymes follow the gene nomenclature and are as follows: Hxk1 = hexokinase, Pgi1 = phosphoglucose isomerase, PFK = phosphofructokinase, a heterooctameric complex composed of four alpha- and four beta-subunits [5], Fba1 = fructose-1,6-bisphosphate aldolase, Tpi1 = triosephosphate isomerase, Zwf1 = glucose-6-phosphate dehydrogenase (“Zwischenferment”), Sol4 = 6-phosphogluconolactonase, Gnd1 = 6-phosphogluconate dehydrogenase, Rki1 = ribosephosphate ketol isomerase, Rpe1 = ribulosephosphate epimerase, Tal1 = transaldolase, Tkl1 = transketolase, and Shb17 = sedoheptulose-1,7-bisphosphatase. Note that for reasons of clarity, the non-glycolytic reaction of Fba1 is depicted in the lower right corner. TCA designates the tricarboxylic acid cycle. Metabolite abbreviations are as follows: Glc = glucose, G6P = glucose-6-phosphate, F6P = fructose-6-phosphate, F1,6P_2_ = fructose-1,6-bisphosphate, DHAP = dihydroxyacetone-3-phosphate, GAP = glyceraldehyde-3-phosphate, Pyr = pyruvate, EtOH = ethanol, 6PGL = 6-phosphogluconolactone, 6PGA = 6-phosphogluconate, Ru5P = ribulose-5-phosphate, Ri5P = ribose-5-phosphate, Xu5P = xylulose-5-phosphate, E4P = erythrose-4-phosphate, S7P = sedoheptulose-7-phosphate, and S17P_2_ = sedoheptulose-1,7-bisphosphate.

In contrast, the milk yeast *Kluyveromyces lactis* is “Crabtree-negative” and largely relies on respiration for its sugar metabolism [3]. In contrast to *S. cerevisiae*, it can grow on glucose even when the glycolytic pathway is blocked by mutations [4,5]. This has been attributed to a higher capacity of the pentose phosphate pathway (PPP) and its participation in glucose degradation (see Figure 1 for a schematic representation and [6] for a recent review). Accordingly, a simultaneous block in glycolysis and the PPP, achieved by an additional deletion of the transaldolase gene, renders the cells incapable of growing on glucose [7].

Despite these initial findings, the genetics of the PPP in *K. lactis* has only sporadically been addressed. Thus, the *ZWF1* gene, encoding glucose-6-phosphate dehydrogenase, which catalyzes the first step of the oxidative part of the PPP, has been cloned and deleted [8]. The *zwf1* deletion grew more slowly than wild-type on rich medium with 2% glucose, a phenotype observed only in some genetic backgrounds for the respective null mutants in *S. cerevisiae* [8,9]. As the NADPH produced is essential for oxidative stress response from yeast to humans, mutants in both yeasts display a distinct hyper-sensitivity towards hydrogen peroxide (reviewed in [6], and [10]). In the course of the studies on the roles of glycolysis and the PPP in sugar degradation in *K. lactis* referred to above, *TAL1* has also been cloned, partially deleted, and biochemically characterized [7]. In those studies, no distinct growth phenotype could be associated with the lack of transaldolase. Yet, specific enzyme activities were found to be approximately five-fold higher than those in *S. cerevisiae*, further supporting the notion of the PPP running at higher capacity in *K. lactis*. Likewise, the specific activity of transketolase was at least three-fold higher in *K. lactis* as compared to *S. cerevisiae*, in which the heterologous *KlTKL1* gene restored activity to the respective deletion, but attempts to construct null mutants in *K. lactis* failed at the time [11].

As this indicated the possibility of the transketolase gene being essential in *K. lactis*, a conditional expression from a tightly regulated promoter would be required for detailed physiological analyses. This has been provided for *S. cerevisiae* with the *tetOFF* system [12]. In brief, it consists of a plasmid-based construct in which the coding sequence for the DNA-binding domain of the bacterial tetracycline repressor is fused to that of the activation domain of the viral VP16 protein (short *tTA* for tet-transcriptional activator). The chimeric transcription factor produced can bind to target *tet* operator sequences (*tetO*_7_) placed between the regulatory elements of the yeast *ADH1* terminator and the *CYC1-TATA* elements. Replacement of target gene promoters by this hybrid promoter will lead to their expression, which can be inhibited by the addition of tetracycline or its derivates, leading to the dissociation of the chimeric transcriptional activator [12]. Consequently, this allows the regulation of gene expression in yeast independent of the carbon source used in the growth medium.

In summary, although some genes coding for enzymes of the PPP have been studied in some detail in *K. lactis*, a systematic investigation has not been provided until now. We here embarked on such an analysis, starting with the construction of deletion mutants individually lacking the enzymes of the PPP. Phenotypic analyses proved three of the genes to be essential. We then concentrated on the analysis of the key transketolase and transaldolase reactions in the non-oxidative part of the pathway. For that purpose, the *tetOFF* system for conditional gene expression was improved, adapted to *K. lactis*, and employed for a broad proteomic analysis of the consequences of their depletion.

## 2. Results

### 2.1. Analyses of PPP

In order to study the PPP in *K. lactis,* we first identified the enzyme encoding genes by homology and synteny analyses. Gene names, systematic designations, the putative enzyme compositions, and the identity of the deduced protein sequences to their *S. cerevisiae* orthologs have been listed, previously [6], and correspond to the reactions shown in Figure 1. PCR-based one-step gene replacements were employed to individually substitute the coding sequences for selectable markers (*ScLEU2* or *kanMX*) in either the diploid recipient strain KHO70 (*MATa*/*MATalpha ura3*/*ura3 leu2*/*leu2 ade2::loxP*/*ADE2 HIS3*/*his3::loxP ku80::loxP*/*ku80::loxP*) or, when the gene was presumably non-essential, with *SpHIS5* in its congenic derivative KHO69-8C (*MATalpha ura3 leu2 his3::loxP ku80::loxP*; [13]). Tetrad analyses were then performed either directly on the diploid strains heterozygous for the deletion, or after crossing their segregants or the original haploid deletions to appropriate wild-type strains.

**Figure 2:**
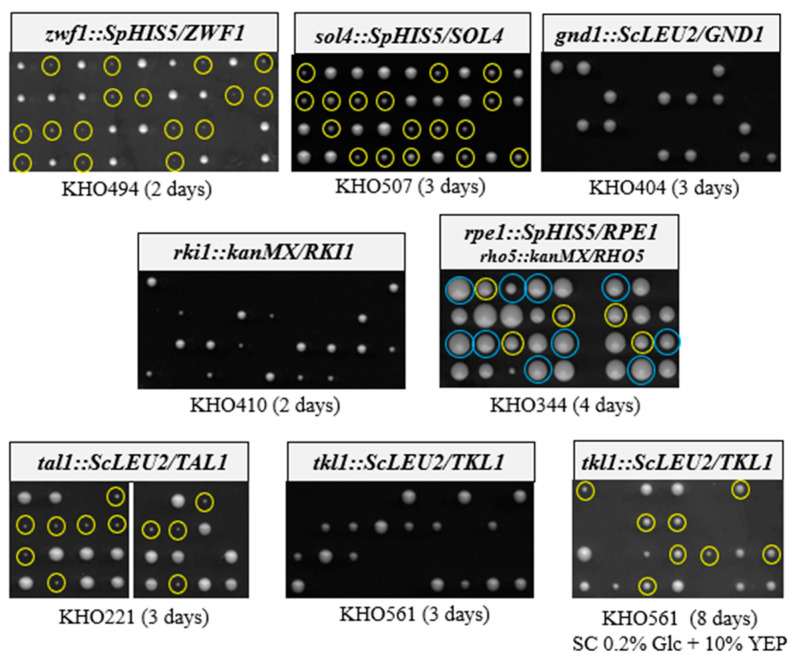
Phenotypes associated with the lack of specific PPP enzymes. Diploid strains heterozygous for the gene deletions indicated were sporulated and subjected to tetrad analyses. Only some representative tetrads are shown from each cross, with a minimum of 18 tetrads analyzed in each case. If not stated otherwise, segregants were obtained on standard YEPD medium with 2% glucose as a carbon source and incubated for the times indicated at 28 °C. Viable segregants carrying the deletion marker are highlighted by yellow circles. Where the deletion proved to be lethal (no circles), the two colony-forming segregants invariably lacked the deletion marker, as expected. Blue circles in the analysis of *rpe1::SpHIS3* also being heterozygous for the *rho5::kanMX* deletion designate the wild-types, and yellow circles the single *rpe1* deletions. Note that in an independent analysis of another heterozygous *rpe1* deletion being wild-type for *RHO5*, the slower growing variants from 10 tetrads analyzed invariably carried the deletion marker, confirming the results shown here. Growth phenotypes were also confirmed in similar analyses employing a different selection marker for the deletion of all genes, with the exception of *RPE1*.

As is evident from Figure 2, all deletions tested on standard rich medium (i.e., yeast extract, peptone with 2% glucose as a carbon source, YEPD) either resulted in a slow growth phenotype as compared to the wild-type segregants (*zwf1Δ*, *sol4Δ*, *rpe1Δ*, and *tal11Δ*) or proved to be lethal, yielding no more than two viable segregants for each tetrad (*gnd1Δ*, *rki1Δ*, and *tkl1Δ*), underlining the importance of the PPP for glucose degradation in *K. lactis*. Several attempts to restore viability to the *tkl1* deletions by supplementing the rich medium with xylose and/or aromatic amino acids and histidine, or with ammonium sulfate, failed. However, a synthetic complete medium with reduced glucose content (0.2%) and containing low concentrations of yeast extract and peptone yielded viable segregants carrying the deletion, although only after prolonged incubation and with a poor overall spore viability (Figure 2). The poor growth of the *tal1* deletions could be slightly improved by reducing the glucose concentration in YEPD to 0.3% and the addition of 5 g/L ammonium sulfate).

### 2.2. Epistasis Analyses Indicate a Physiological Control by Metabolic Intermediates

**Figure 3:**
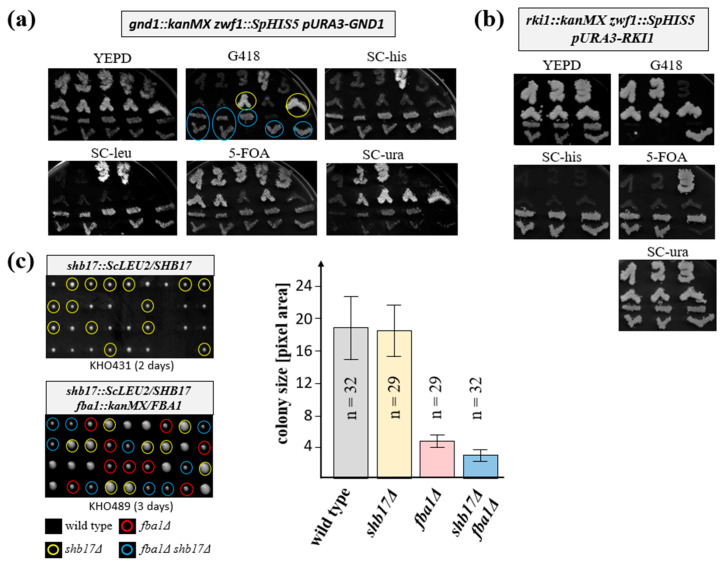
Epistasis analyses of different PPP null mutant combinations (**a**,**b**). For the preparation of master plates, segregants were picked according to their colony sizes, starting with the larger colonies and followed by the smaller ones. Numbers written with the first segregants reflect the tetrad number from each cross. Master plates were generated on YEPD and incubated overnight at 28 °C, before replica-plating onto the indicated media. Images were taken after 1–2 days of incubation at 28 °C. Strains employed were KHO528 (**a**) and KHO526 (**b**). Yellow circles in (**a**) highlight *gnd1* deletions carrying the plasmid, and blue circles the double deletions with *zwf1*. (**c**) The strains designated below each plate were sporulated, subjected to tetrad analysis on YEPD plates, and incubated for the times indicated at 28 °C. Colony sizes were assessed by determination of the pixel areas using the ImageJ program 1.53c. Columns show the mean colony sizes with error bars indicating the standard deviations from the total number of colonies (n) examined for each genotype.

Early mutant studies in *S. cerevisiae* showed that the accumulation of 6-phosphogluconate can be toxic for cells lacking the subsequent dehydrogenase reaction [14]. We reasoned that this may also be the case in *K. lactis*, explaining the lethality of the *gnd1* deletion. Therefore, double null mutants with the *zwf1* deletion, encoding the preceding step of the glucose-6-phosphate dehydrogenase (Figure 1), were obtained by crossing a *gnd1Δ* strain complemented by a plasmid-borne wild-type *GND1* gene with an appropriate *zwf1Δ* segregant, followed by tetrad analysis. Representative tetrads shown in Figure 3a demonstrate that *gnd1* deletions can only survive if they carry the complementing plasmid, as demonstrated by their inability to grow on the counter-selective 5-FOA medium (i.e., where cells with a wild-type *URA3* gene used as the plasmid-marker will die). However, segregants carrying the double deletion (*zwf1Δ gnd1Δ*) produced colonies even in the absence of the plasmid as indicated by their uracil auxotrophy. This is further supported by the fact that all double deletions originally carrying the plasmid are able to grow upon counter-selection, i.e., can lose the wild-type *GND1* allele. Together, these findings strongly suggest that preventing the accumulation of 6-phosphogluconate indeed can rescue the lethality of the *gnd1* deletion.

Encouraged by these results, we performed a similar epistasis analysis with the non-viable *rki1* deletion complemented by a plasmid-borne wild-type gene, again crossing it with a *zwf1* deletion (Figure 3b). Clearly, segregants carrying the *rki1* deletion were only viable if they carried the complementing plasmid, irrespective of if they also carried the *zwf1* deletion. Accordingly, none of the *rki1* segregants grew on the counter-selective 5-FOA medium, indicating that the loss of viability in strains lacking the ribosephosphate ketol isomerase cannot be attributed to a tailback accumulation of 6-phosphogluconate.

We then addressed a question that can be more easily approached by epistasis analysis in *K. lactis* than in the common model yeast *S. cerevisiae*: A sedoheptulose-1,7-bisphosphatase (Shb17) has been proposed to function in the replenishment of the non-oxidative PPP in *S. cerevisiae*, but apart from an accumulation of sedoheptulose and octulosephosphates, its lack was not associated with a distinct phenotype [15]. The authors proposed the production of the sugar bisphosphate from a side-reaction of the glycolytic fructose-1,6-bisphosphate aldolase (Fba1) as depicted in the lower right-hand corner of Figure 1. Since in contrast to *S. cerevisiae K. lactis,* strains deleted for *FBA1* can still grow on glucose, albeit more slowly than the wild-type [16], we decided to investigate these reactions more closely. For this purpose, an epistasis analysis was performed by studying the combination of the two deletions, *shb17Δ* and *fba1Δ*. As shown in Figure 3c, while *shb17Δ* segregants did not show a significant growth defect on rich medium as compared to the wild-type, the slow-growth phenotype of the *fba1Δ* segregants was further aggravated in the double deletions. This suggests an important but not essential role for the replenishment reaction in the physiology of *K. lactis*.

### 2.3. Proteome Analyses After Depletion of Transketolase or Transaldolase Indicate a Relation to Mitochondrial Functions

Since the reactions catalyzed by Tkl1 and Tal1 are crucial for the exchange of metabolites between glycolysis and the PPP in general (Figure 1), and in particular for glucose degradation in *K. lactis* as indicated by the severe growth defects of the deletion mutants shown above, we decided to further concentrate on these two enzymes.

**Figure 4:**
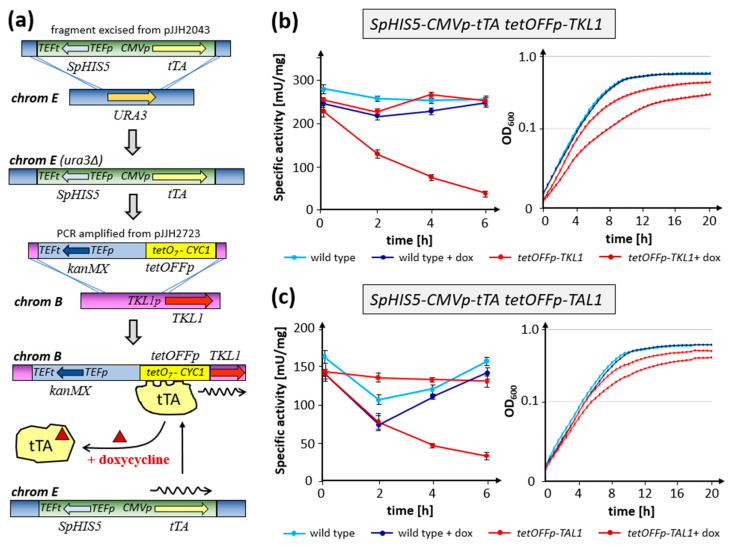
Establishment of the *tetOFF* system for conditional expression of transketolase and transaldolase in *K. lactis*. (**a**) Genomic integration of the *tTA* expression cassette and of a selectable *tetOFF* promoter construct for PCR-based one-step substitutions. The *TKL1* promoter is depicted as an example for conditional expression of any desired target gene. (**b**) Kinetics of transketolase depletion followed by determination of specific enzyme activities (**left**) and effect on growth (**right**). (**c**) Kinetics of transaldolase depletion followed by determination of specific enzyme activities (**left**) and effect on growth (**right**) on synthetic complete medium (SCD).

For this purpose, a method to tightly control the respective gene expressions had to be established in *K. lactis*, which ideally should work independent of the carbon source employed. We chose the *tetOFF* system, as it is widely used in different model organisms and had been previously adapted in the closely related yeast *Kluyveromyces marxianus* [17,18]. To facilitate the use of this system on a broader scale in which only the promoter of interest has to be replaced by the *tet* operator construct, the native *URA3* gene of *K. lactis* was stably replaced in a first step by an expression cassette for the hybrid *tet* repressor/activator (*tTA*) as outlined in Figure 4a.

A synthetic *tetOFF* promoter sequence was then cloned downstream of the *kanMX* resistance cassette in a plasmid serving as a template for PCR amplification. Fragments thus generated direct the replacement of any given promoter sequence, exemplified by the *TKL1* promoter in Figure 4a, through homologous recombination with the flanking sequences introduced by the appropriate choice of oligonucleotide primers. Using the strain carrying the *tTA* construct as a recipient, the target gene is thus expressed under standard growth conditions without further selection. However, it can then be repressed by the addition of tetracycline, or its analog doxycycline to the growth medium. If required, the *ku80* deletion can subsequently be eliminated by a simple back cross to a wild-type strain followed by tetrad analysis, as can any other genetic background be introduced, such as the combination with another gene deletion.

Using this strategy, strains with conditional expression of either *TKL1* or *TAL1* were obtained. As expected, the addition of doxycycline to cells growing in rich medium resulted in a drastic decrease in the respective enzyme activities within six hours of incubation, but not in the controls without the drug or the wild-type expressing the genes from their native promoters (Figure 4b,c). Note that the initial but transient drop in the specific activity of transaldolase displayed by the wild-type was also observed in other controls, and may thus be attributed to an adaptation to the fresh medium. Consistent with the decrease in enzyme activities, growth of the respective strains slowed down under the influence of doxycycline, as opposed to the wild-type and the cultures without the drug (Figure 4b,c). This effect was more pronounced in strains depleted for transketolase, i.e., where the gene deletion appeared to be lethal in rich medium, than in those depleted for transaldolase, for which deletions remained viable as shown above.

**Figure 5:**
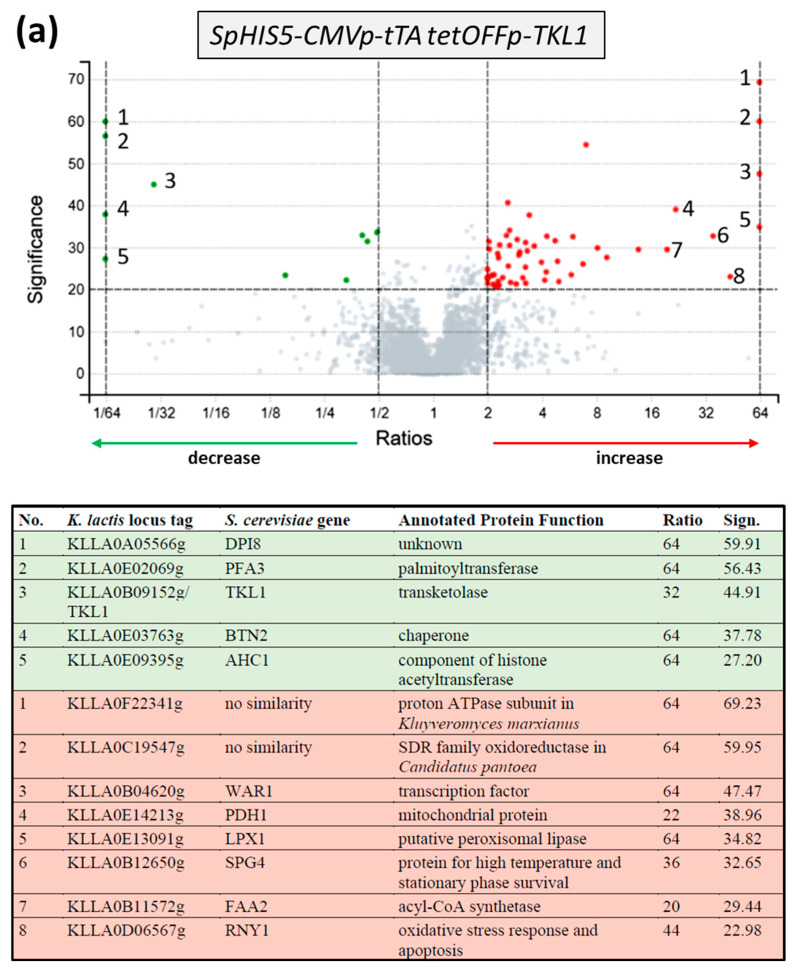

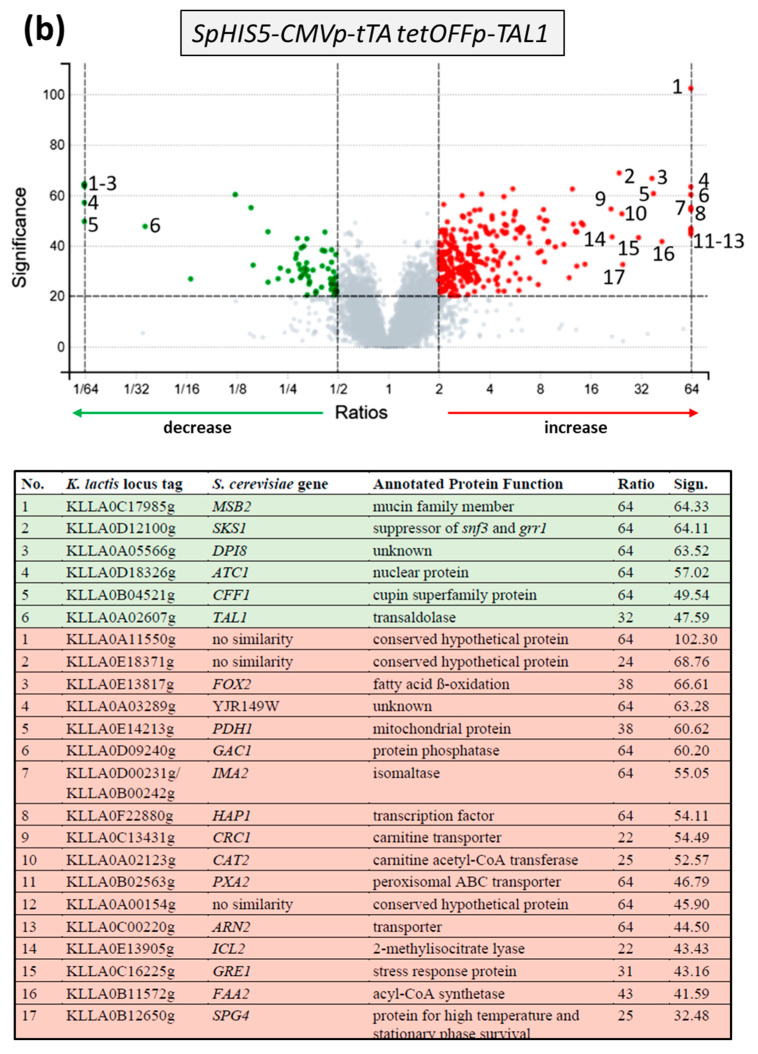
Comparative proteome analyses after depletion of transketolase (**a**) or transaldolase (**b**). Volcano plots show the decrease (green dots) and increase (red dots) in proteins upon incubation of the strains KHO382-1B (**a**) and KHO551-3B (**b**) with the relevant genotypes depicted in the gray shaded bars with doxycycline to inhibit gene expression. As indicated, cut-offs were chosen at a factor of 2 relative to the untreated control and at a significance of 20. Tables below the plots are numbered according to the plots, with downregulation on pale green and upregulation on a pale red background. Peptides identified by mass spectrometry were assigned using basically the annotations in the *Saccharomyces* genome database (https://www.yeastgenome.org; last accessed on 20 December 2024) and annotated to the *K. lactis* genes in the KEGG database (https://www.genome.jp; last accessed on 20 December 2024).

In order to gain some insight into the physiological consequences of transketolase or transaldolase depletion, the *tetOFF* strains were then subjected to an unbiased, label-free proteome analysis. Therefore, samples were prepared from cultures growing in synthetic complete medium in the presence or absence of doxycycline for at least 20 h, harvested during logarithmic growth, and subjected to mass spectrometry analysis (see Section 4 for further details on growth conditions and technical parameters). Confirming the results of the enzymatic determinations presented above, concentrations of Tkl1 and Tal1 were drastically reduced in the respective strains (*tetOFFp-TKL1* and *tetOFFp-TAL1*, both approximately 32-fold) when exposed to doxycycline as compared to the controls grown without the drug. A large number of other proteins was either up- or downregulated in the two strains, as is evident from the volcano plots in Figure 5a,b. Proteins showing a more than 16-fold change in concentration in either direction are listed below each volcano plot.

In total, depletion of transketolase led to a significant increase in the amount of 55 proteins, compared to a decrease in 10 others (Supplementary Table S1). The effect of transaldolase depletion was even stronger, with 260 proteins increasing and 61 decreasing as compared to the controls not exposed to doxycycline (Supplementary Table S1). As the vast majority of differentially detected proteins thus appeared to be increased upon depletion of transketolase or transaldolase, we concentrated only on those proteins with an at least two-fold change and assigned them to functional categories, which were manually curated for metabolic pathways (Table 1).

A pronounced increase upon depletion of both transketolase or transaldolase was observed for proteins of the fatty acid ß-oxidation pathway located in peroxisomes and related reactions in the transport to and the degradation of acetyl-CoA in mitochondria (i.e., the peroxisomal proteins, the carnitine shuttle and the methylcitrate pathway listed in Table 1). In addition, an increase in the concentration of stress-related proteins was observed upon depletion of the PPP enzymes. This may be attributed to the cells’ attempt to compensate for the expected shortage in energy production from glucose caused by the impairment of the PPP, as will be elaborated on in the discussion.

Interestingly, several cell wall glycoproteins (Sed1, Cis3, Gas1, Scw10, Toh1, Pir1, Ncw2, Ptp2, Skt5, and Cwp1) also showed much higher levels upon depletion of transaldolase compared to control cells, but were not differentially regulated if transketolase was depleted (Supplementary Table S1). Two key enzymes of gluconeogenesis, the phosphoenolpyruvate carboxykinase Pck1 and the fructose-1,6-bisphosphatase Fbp1, were also increased roughly by a factor of 3.5 upon transaldolase—but not transketolase—depletion (Supplementary Table S1).

### 2.4. Heterologous Expression of TKL and TAL Genes from Yeasts and Humans

**Figure 6:**
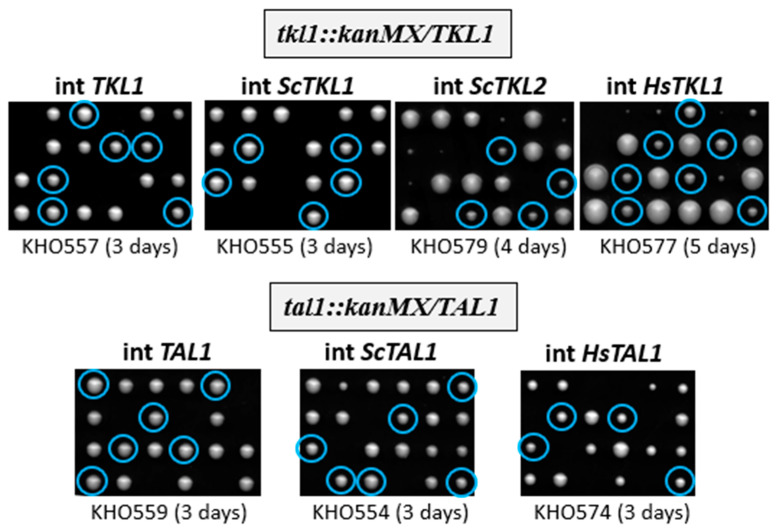
Tetrad analyses of strains heterozygous for the *tkl1* or *tal1* deletion carrying complementing genes integrated at the *leu2* locus. Designations of the strains employed and times of incubation at 28 °C on standard YEPD medium are indicated below each plate. For KHO577 and KHO579, a rich medium containing only 0.3% glucose was used for tetrad analysis, additionally supplemented with ammonium sulfate and uracil. The very small colonies not circled represent *tkl1* deletions not carrying the integrated *ScTKL2* allele. Blue circles designate the colonies carrying the integrated constructs in the background of the respective deletion.

Finally, as transketolase and transaldolase deficiencies are associated with severe diseases, we wondered whether the *K. lactis* deletions described above could be employed for functional studies of the homologues from other organisms, including humans. Therefore, the two paralogues encoding transketolase isoforms in *S. cerevisiae* (*ScTKL1*, *ScTKL2*), as well as the gene for transaldolase (*ScTAL1*) were cloned from the host genome including their flanking sequences, i.e., under the control of their native promoters. For the human homologues, custom gene synthesis was employed to generate the coding sequences of *HsTKL1* and *HsTAL1* in the *S. cerevisiae* codon usage. These were cloned under the control of a tailored *ScPFK2* promoter to drive gene expression in *K. lactis*. All constructs were obtained in the backbone of a *K. lactis* vector (pJJH2600L) and integrated at the *leu2* locus by linearization prior to transformation of the recipient strains and selection for leucine prototrophy to avoid variations caused by fluctuating plasmid copy numbers. Diploid recipient strains heterozygous for the respective deletions, Kl1323 (*tkl1::kanMX*/*TKL1*) and Kl1324 (*tal1::kanMX*/*TAL1*), and homozygous for the *leu2* mutation were employed throughout and subjected to tetrad analyses (Figure 6) to yield segregants with the heterologous alleles expressed in the deletion background (e.g., with the genotype *tkl1::kanMX leu2::LEU2-ScTKL1,* etc.). The native *K. lactis TKL1* and *TAL1* genes were also cloned and integrated at the *leu2* locus and served as positive controls.

A representative segregant exclusively expressing an integrated allele was chosen for each type from such tetrad analyses, crude extracts were prepared from cells grown in rich medium, and specific transketolase and transaldolase activities were determined (Table 2). As expected, transketolase and transaldolase activities were restored approximately to wild-type levels in strains with the native alleles (compare CBS2359 to KBO23-2B and KBO28-5B, respectively), demonstrating that ectopic integration does not significantly interfere with gene expression.

Integration of the heterologous *ScTKL1* gene (KBO22-4A) complemented the *K. lactis* deletion, but yielded only approximately half the specific transketolase activity as compared to the wild-type or the integrant with the native allele (Table 1). Interestingly, the strain carrying the paralogue *ScTKL2* also rescued the lethal phenotype of the *K. lactis tkl1* deletion, as demonstrated by the generation of viable segregants in the tetrad analysis of the recipient strain (Figure 6). Despite this complementation in vivo, in vitro measurable transketolase activity remained below the level of detection. In contrast, expression of the human isoform (KBO25-2C) yielded approximately 25% of the wild-type activity, demonstrating that human homologues, and presumably their mutant alleles, can be studied in this genetic background of *K. lactis*.

Likewise, heterologous expression of the yeast *ScTAL1* gene and that of the human homologue in the *tal1* deletion background restored enzyme activity to the respective segregants, albeit to only 25% (KBO27-2A) and 15% (KBO30-2A) of the wild-type level, respectively (Table 1). Since *tal1* deletions are viable, they could be grown for the preparation of crude extracts and show no detectable transaldolase activity, as expected.

## 3. Discussion

The genetics of the pentose phosphate pathway in the dairy yeast *Kluyveromyces lactis* was studied in this work since (i) the PPP is much more important for sugar degradation in this yeast as compared to the well-studied baker’s yeast *Saccharomyces cerevisiae* [6], and (ii) in contrast to the latter, the genome of *K. lactis* was not duplicated in the course of evolution [38] so that in most cases the deletion of one instead of two or more paralogous genes is sufficient to eliminate enzyme activity and study the associated physiological phenotypes. However, it is as amenable to both classical and molecular genetic techniques as the standard model yeast *S. cerevisiae* [39].

As we previously established a congenic strain series with a set of suitable selection markers [13], phenotypes of the deletions obtained herein are most likely attributed to the gene in question, rather than variations in the genetic background. Consistent with the shared role of the PPP with glycolysis in glucose degradation of *K. lactis*, we found that all strains lacking an enzyme within the standard textbook pathway were impaired to various degrees for growth on this carbon source. Of special interest were the genes that appeared to be essential, i.e., those that did not produce viable colonies on standard rich medium. For *gnd2Δ,* it resembled the lethality of a double deletion of the two *S. cerevisiae* paralogues, which had been attributed to a toxic accumulation of 6-phosphogluconate [14]. In accordance, an additional deletion of the gene for glucose-6-phosphate dehydrogenase (*zwf1Δ*) in *K. lactis* prevented the formation of the toxic metabolite, rescued these mutants, and produced viable progeny. It should be noted that the slow-growth phenotype of single *zwf1Δ* strains is not alleviated in the double mutants (*zwf1Δ gnd1Δ*), and is observed in only a few genetic backgrounds in *S. cerevisiae* strains lacking glucose-6-phosphate dehydrogenase (see [9], and references therein). In *K. lactis*, the enzyme has been extensively studied and the slow-growth phenotype of deletion mutants was detected and discussed previously [8,40]. We assume that the growth impairment of strains lacking the 6-phosphoglucono lactonase (*sol4Δ)* can be equally attributed to a lower flux of glucose-6-phosphate into the PPP in *K. lactis*.

Interestingly, the toxic accumulation of 6-phosphogluconate only explains the impact on the initial steps of the oxidative part of the PPP in *K. lactis*, since the lethality of *rki1Δ* strains, lacking the ribose-5-phosphate ketol isomerase, cannot be alleviated in combination with *zwf1Δ*. *RKI1* was also found to be an essential gene in *S. cerevisiae* and proposed to serve another, yet unidentified function apart from the catalysis of the PPP reaction, since the lack of other enzymes of the non-oxidative part of the pathway did not impair viability [41]. This was supported by the finding that a viable point mutation, *RKI1^R189K^*, was isolated for its pyridoxine auxotrophy in *S. cerevisiae*, which retained only 0.6% of the wild-type enzymatic activity [42]. Additionally similar to *S. cerevisiae*, *K. lactis* strains lacking the ribulose-5-phosphate epimerase (*rpe1Δ*) were perfectly viable and indeed showed the least, but still consistent, impairment of colony sizes of the segregants upon tetrad analysis compared to all other mutants tested herein.

In contrast, *TKL1* appeared to be essential at first sight, as no viable progeny was obtained from tetrad analyses of heterozygous deletion strains on standard rich medium. This explains why previous attempts to delete the gene in *K. lactis* failed [11] and stands in contrast to the *S. cerevisiae* mutants, where deletion of the major *ScTKL1* gene did not impair growth, and in combination with deletion of its paralogue, *ScTKL2*, only showed auxotrophy for aromatic amino acids [43,44]. Viability to the *K. lactis* deletion could not be restored by the simple addition of these amino acids, but only after prolonged incubation on synthetic complete medium with reduced glucose concentrations (0.2%) and supplemented with minor amounts of yeast extract and peptone. We consider these conditions too time-consuming and non-practical for experimental purposes, and the *TKL1* gene to be semi-essential. Yet, the viability upon reduction in the external sugar concentration may indicate the accumulation of toxic intermediary metabolites of the PPP under standard growth conditions in the *tkl1Δ* deletion, which may be prevented by reducing the influx from glycolytic intermediates. Of note, expression of the *TKL2* gene from *S. cerevisiae* in the *tkl1Δ* background of *K. lactis* restored growth to some extent, but lacks detectable transketolase activity. This supports the conclusion drawn from overexpression studies in *S. cerevisiae* that Tkl2 confers sufficient enzyme activity in vivo to complement the lack of Tkl1 [43].

The notion of toxic intermediates accumulating at high external glucose concentrations in PPP mutants is supported by the observation that the slow-growth phenotype of segregants lacking transaldolase is partially alleviated on rich medium by reducing the glucose concentration to 0.3%. That growth impairment of *tal1Δ* deletions that was not observed in earlier studies [7] may be attributed either to variations in the medium components or to the fact that the coding sequence could only be partially deleted at the time, in contrast to the complete deletions reported here.

While more recent metabolome analyses in *S. cerevisiae* revealed the presence of a sedoheptulose-1,7-bisphosphatase, deletion of the *SHB17* gene, conserved amongst yeast species, was not associated with a growth phenotype, but only resulted in an increase in intracellular sedoheptulose and octulose phosphates [15]. The authors proposed that the substrates for this phosphatase are produced by a side reaction of the glycolytic fructose-1,6-bisphosphate aldolase. This notion was hard to test in *S. cerevisiae* by genetic analyses, since the *fba1* deletion grows extremely poor [45]. On the other hand, we showed that the respective deletion in *K. lactis* is sufficiently viable to permit epistasis analysis with the *shb17* deletion constructed herein [16]. Indeed, while growth of segregants upon tetrad analysis was not significantly reduced for *shb17Δ* strains, the slow-growth phenotype of *fba1Δ* strains was exacerbated in the *fba1Δ shb17Δ* double deletions. This strongly indicates that the sedoheptulose-1,7-bisphosphatase is produced in *K. lactis* and serves a relevant physiological function dependent on the presence of the aldolase, as suggested in the initial discovery [15].

Despite the advantage of mutants with defects in glycolysis being viable, the investigation of essential gene functions in *K. lactis*, especially with regard to carbohydrate metabolism, is strongly impaired by the lack of a sugar-independent conditional expression system. We therefore adopted and improved the plasmid-based *tetOFF* system previously shown to work in the closely related *Kluyveromyces marxianus* [17]. Substitution of the native *URA3* gene at its chromosomal locus by a modified *tet* repressor/activator *tTA* construct allows (i) for stable expression of the transcription factor on non-selective media, and (ii) the identification of strains carrying the construct in tetrad analyses, conferring uracil auxotrophy and histidine prototrophy. In addition, a plasmid was constructed that can serve as a template for PCR-mediated amplification of the target *tetOFF* promoter for one-step promoter substitutions with a flanking selection marker. This will be suitable to control any target gene, being expressed under standard growth conditions but repressed by doxycycline addition.

Proof-of-principle was then provided by controlling expression of *TKL1* and *TAL1* by using this system. Both the reduction in the respective enzyme activities and the decrease in growth rates upon addition of doxycycline confirmed the expected regulation. However, enzyme activities did not drop below detectable levels, nor did the proteins escape detection by mass spectrometry after prolonged growth in the presence of doxycycline. Rather, protein levels amounted to approximately 3% of those of the non-treated controls. Consistent with this, *tkl1* deletion segregants continued to grow on plates containing doxycycline after repeated replica plating, indicating (i) a residual level of gene expression and (ii) that less than 3% of the wild-type transketolase level is sufficient to confer viability. Unpublished results from our laboratory show that viability is also retained under repression conditions when the promoters of several other essential genes are replaced by the *tetOFF* construct.

**Figure 7:**
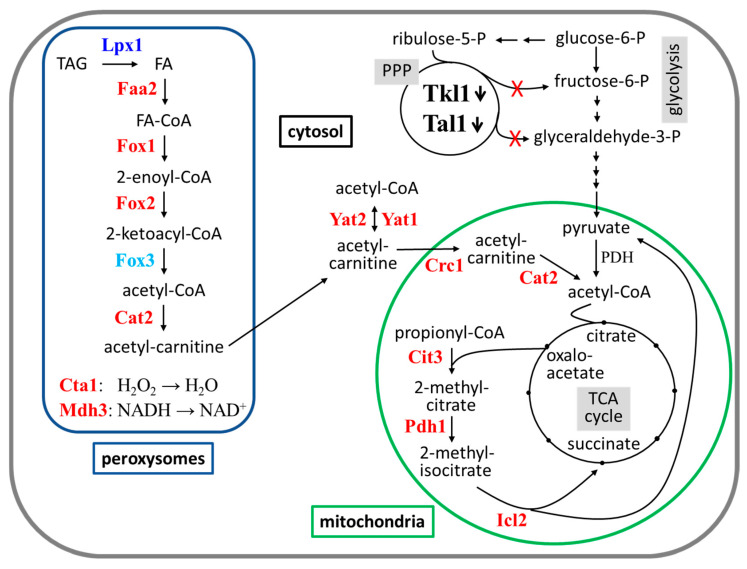
Overview on the connection between peroxisomal fatty acid degradation and mitochondrial turnover of acetyl-CoA with the pathways for glucose degradation in *K. lactis* (adapted from [46,47,48]). The reactions corresponding to the enzymes upregulated upon depletion of transketolase or transaldolase are shown with regard to their intracellular distribution in peroxisomes (dark blue line), mitochondria (green line), or the cytosol (compare Table 1 for functions and fold-changes in protein concentrations). Enzyme names in red designate those increased upon depletion of both transketolase or transaldolase. Lpx1 (dark blue) is only increased if transketolase is depleted, Fox3 (light blue) only upon transaldolase depletion. Note that Yat1 localization at the outer mitochondrial membrane is not shown here for reasons of clarity in presentation. Depicted metabolic pathways (gray shaded) include the pentose phosphate pathway (PPP with depletion of the two key enzymes indicated by the downward arrows), glycolysis, and the tricarboxylic acid cycle (TCA). Other abbreviations are as follows: TAG = triacylglycerol, FA = fatty acid, and PDH = pyruvate dehydrogenase complex.

Nevertheless, a comparative proteome analysis by mass spectrometry of the strains incubated with doxycycline versus the controls growing without the drug yielded significant changes for 65 proteins upon transketolase depletion and for 321 proteins upon transaldolase depletion. As expected, transketolase and transaldolase were amongst the proteins drastically reduced, providing an internal control. Functional categorization revealed a predominance of proteins being increased upon depletion of both enzymes in the processes of fatty acid oxidation, carnitine shuttle, and mitochondrial proteins (Figure 7).

One may speculate that these enzymes are specifically increased to compensate for a shortage in energy production. As stated above, glycolysis and the PPP seem to equally contribute to glucose degradation in *K. lactis*. Diminishing the flux through the PPP by depletion of its key enzymes may thus slow down the production of pyruvate, as the common intermediates fructose-6-phosphate and glyceraldehyde-3-phosphate will have to be exclusively provided by the glycolytic reactions. Consequently, less acetyl-CoA will be available as a fuel for the tricarboxylic acid cycle, and ultimately ATP generation by respiration. As shown in Figure 7, this could be provided by the degradation of fatty acids, as well as the 2-methylcitrate pathway. Thus, the physiology of *K. lactis* would reflect that of humans, as we also use fatty acids as an energy source when starved for carbohydrates (see [49] and references therein).

The increased activity of the fatty acid ß-oxidation pathway could also explain the observed increase in the stress-induced proteins listed in Table 1. Thus, peroxisomes are crucial in maintaining homeostasis upon oxidative stress and hydrogen peroxide is a by-product of fatty acid degradation at the level of the Fox1 reaction [50,51]. As ace-tyl-CoA thereby produced is further transported into mitochondria by the carnitine shuttle and then enters the TCA cycle and respiration, more reactive oxygen species are expected to be produced in this organelle [52]. Together, this would trigger the oxidative stress response pathway in *K. lactis* [53]. 

Finally, although only observed upon depletion of transaldolase and not of trans-ketolase, we detected an increase in the amount of ten different cell wall proteins. This is highly significant, as only 22 covalently linked cell wall proteins have been described in *K. lactis* [54]. Together with the observation that two key enzymes of gluconeogenesis (phosphoenolpyruvate carboxykinase and fructose-1,6-bisphosphatase) are also in-creased in Tal1-depleted cells, we believe that they serve to strengthen the cell wall and contribute to the resistance to adverse environmental conditions. Since the cell wall proteins are highly mannosylated and fructose-6-phosphate serves as a precursor for this sugar, gluconeogenesis may compensate for the diminished supply caused by the downregulation in the PPP. 

In addition to gathering the proteome data, we employed the *tkl1* and *tal1* deletion mutants for the expression of the homologous genes from baker’s yeast and humans. As shown in the final section of results (Section 2.4) they complemented the growth defects and re-stored the respective enzymatic activities, albeit at a lower level than the native genes. This provides evidence that human disease alleles of transketolase and transaldolase genes from patients may be expressed and investigated in *K. lactis* as a model system, given the closer resemblance of its physiological to mammalian cell metabolism, especially with regard to the PPP [6,10].

## 4. Materials and Methods

### 4.1. Media, Strains, and Culture Conditions

Rich media were based on yeast extract (1% *w*/*v*), peptone (2% *w*/*v*) from Difco Laboratories Inc., Detroit, MI, USA, with glucose (2% *w*/*v*) as a carbon source (YEPD). Variations in the nature or concentration of the carbon source are indicated where applicable. Synthetic media were based on 0.67% (*w*/*v*) Difco yeast nitrogen base with ammonium sulfate and 2% glucose (*w*/*v*) as the carbon source (SCD) with the addition of amino acids and bases using a mixture provided by MP Biomedicals (Eschwege, Germany; CSM-His-Leu-Trp-Ura) supplemented with histidine, leucine, tryptophane, or uracil as required for selection of plasmids or deletion markers. Additionally, 100 mg/L G418 were used for selection of the dominant *kanMX* marker. For the preparation of solid media, 1.5% (*w*/*v*) agar was added prior to sterilization. Yeasts were incubated at 28 °C, with constant agitation at 180 rpm for liquid cultures.

*K. lactis* strains employed in this work are listed in Table 3.

For work with *E. coli*, strain DH5α (Invitrogen, Karlsruhe, Germany) was used, grown at 37 °C in LB medium (0.5% yeast extract, 1% Bacto peptone, 0.5% sodium chloride, all *w*/*v*), and supplemented with ampicillin (50 mg/L) or kanamycin (25 mg/L) for plasmid selection.

### 4.2. Construction of Deletion Mutants, Integration of Heterologous Genes and Establishment of the tetOFF System

For transformation of *K. lactis*, a modified freeze method was used as described [55].

Deletions were either constructed by PCR-based one-step gene replacements [56], with primers generating 40–50 bp of homology flanking the genomic target sequences, or by introducing larger flanking sequences homologous to the target gene by in vivo recombination in *S. cerevisiae* strain DHD5 [55]. Yeast transformants were grown on selective medium, and plasmids were isolated and amplified in *E. coli*. The deletion cassettes were than excised from the plasmid DNA by restriction digests and introduced into the *K. lactis* recipient strains selecting for homologous recombinants. Thereby, open reading frames of the target genes were entirely replaced by selection markers amplified from plasmids described in [57] or [13]. Correct deletions were verified by PCR using appropriate primer pairs. Maps and sequences of the deleted loci and of oligonucleotides employed are available upon request.

Integrative plasmids for expression of heterologous genes were based on pJJH2600L, in which the *LEU2* gene from *K. lactis* was cloned into pUK1921 [58], conferring kanamycin resistance in *E. coli*. Derivatives carrying a heterologous gene were either linearized with KpnI or with XcmI prior to transformation of the recipient strains to direct integration at the *leu2* locus. Correct integration was confirmed by three independent PCR reactions each, with amplification of the entire insertion, or generating either 5’ or 3’ specific fragments with primers binding within the integrated sequences. Maps and sequences of the respective integrants are available upon request.

For establishment of a mitotically stable *tetOFF* system in *K. lactis*, the coding sequence for the chimeric tTA transactivator under the control of the CMV promoter was subcloned from pCM189 [12] between the flanking sequences of the *KlURA3* gene to yield pJJH2043, which was then used for genomic substitution as depicted in Figure 4. The *tetO_7_* sequence flanked by the *ADH1* terminator and the *CYC1* TATA elements as described in [12] was obtained from custom DNA synthesis by ThermoFisher Scientific (Bremen, Germany) and subcloned downstream of the *kanMX* selectable marker of pUG6 [57] to yield pJJH2723. The latter serves as a template to generate PCR-based promoter substitution cassettes as also exemplified in Figure 4. 

### 4.3. Growth Curves

Cells were pregrown overnight in 5 mL of YEPD at 28 °C with shaking at 180 rpm, diluted to an OD_600_ of 0.3 in fresh SCD medium, and allowed to grow for another 5 h. They were adjusted to OD_600_ of 0.1 in SCD and three technical replicates of each strain were applied to a microtiter plate. Growth curves were obtained in 100 µL cultures in 96-well plates in SCD medium and recorded in a Varioscan Lux plate reader (Thermo Scientific, Bremen, Germany). For statistical analyses and preparation of graphics, Rv 4.1.0 and R Studio v 1.41717 were used [59].

### 4.4. Tetrad and Epistasis Analyses

Parental *K. lactis* strains were streaked out against each other on 3% malt agar plates. Plates were then incubated for 2 days at 28 °C to allow for mating and diploids were selected for complementation of suitable auxotrophic markers by replica-plating onto appropriate selection media. After growth for 8 h in 3 mL YEPD to an OD_600_ of approximately 0.5, cells were sporulated on plates with 1% potassium acetate and subjected to tetrad analyses on YEPD plates using a Singer MSM400 micromanipulator (Singer Instruments, Somerset, UK). Plates were incubated for 2–5 days at 28 °C as required for the specific mutant combinations and scanned for documentation. The images were adjusted for brightness and contrast with the same settings for the entire plate. For determination of colony sizes, the ImageJ program 1.53c was employed with the analyze particles function. At least 18 tetrads were separated for each mutant combination and the genotypes were assigned from marker analyses.

### 4.5. Enzyme Assays

Cultures for enzymatic determinations were grown overnight in rich medium (YEPD), inoculated in fresh medium to an OD600 of 1.0, and grown for another 5 h with shaking at 180 rpm at 28 °C. Cells were then harvested by centrifugation and stored at −20 °C until use. Crude extracts were prepared by breaking of cells with glass beads and centrifugation. Transketolase and transaldolase activities were determined in 50 mM glycylglycine buffer, pH 7.5 by coupled enzyme assay. The test mix contained one of the two substrates for each enzyme (0.5 mM xylulose-5-phosphate for transketolase and 2.5 mM erythrose-4-phosphate for transaldolase). After recording the baseline upon addition of crude extract, the second substrate was added to start the reaction (0.4 mM erythrose-4-phosphate for transketolase and 2 mM fructose-6-phosphate for transaldolase). For transketolase tests, the assay mixture also contained 10 mM magnesium chloride, 0.2 mM thiamine pyrophosphate, 3 mM calcium chloride, and 0.15 mM dithiothreitol. The products of both enzyme reactions were directed to the oxidation of 0.2 mM NADH with the help of auxiliary enzymes (1U each of glycerol-3-phosphate dehydrogenase and triosephosphate isomerase), and the decrease at 340 nm was followed in a DU800 spectrophotometer (Beckman-Coulter, Krefeld, Germany) at 30 °C. Specific activities were determined by normalizing to the total protein concentration in the crude extracts, determined by the Biuret reaction, using bovine serum albumin as a standard [60]. Mean specific activities and standard deviations were determined from three biological and three technical replicates using standard settings in the Microsoft Excel program.

### 4.6. Proteome Analyses

Proteomes were obtained from mass spectrometry analyses. Therefore, yeast cells were grown in 25 mL SCD in the presence or absence of 5 µg/L doxycycline, which were inoculated from a fresh overnight culture grown under the same conditions to an OD600 of 0.2 and allowed to grow for another 6 h at 28 °C and shaking at 180 rpm. Samples were extracted using the iST kit according to the instructions of the manufacturer (Preomics GmbH, Martinsried, Germany). Mass spectrometry using label-free quantification (LFQ) was performed at the Mass Spectrometry Equipment Center of the Department of Biology/Chemistry at the “CellNanOS” research center of the University of Osnabrück. To prepare samples for quantification, dried peptides were resuspended in 10 µL LC-Load buffer and 2 µL were applied to reversed-phase chromatography on a Thermo Ultimate 3000 RSLC-nano system connected to a TimsTOF HT mass spectrometer (Bruker Corporation, Bremen, Germany) through a captive spray ion source. Peptides were separated on an Aurora Gen3 C18 column (25 cm × 75 µm × 1.6 µm) with CSI emitter (Ionoptics, Collingwood, Australia) at a temperature of 40° C. Peptides from the column were eluted via a linear gradient of acetonitrile from 10 to 35% in 0.1% formic acid (*v*/*v*) for 44 min at a constant flow rate of 300 nL/min following a 7 min increase to 50%, and finally, 4 min to reach 85% buffer B. Eluted peptides were then directly electro sprayed into the mass spectrometer at an electrospray voltage of 1.5 kV and 3 L/min dry gas. 

The MS settings of the timsTOF were adjusted to positive ion polarity with a MS range from 100 to 1700 *m*/*z*. The scan mode was set to PASEF. The ion mobility was ramped from 0.7 Vs/cm^2^ to 1.5 in 100 ms. The accumulation time was also set to 100 ms. Addtionally, 10 PASEF ramps per cycle resulted in a duty cycle time of 1.17 s. The target intensity was adjusted to 14,000, and the intensity threshold to 1200. The dynamic exclusion time was set to 0.4 min to avoid repeated scanning of the precursor ions, their charge state was limited from 0 to 5. The resulting data were analyzed with PeaksOnline (BSI, Canada) version 11, employing the corresponding Yeast FASTA databases. Precursors were ranging from 600 to 6000 Da. As modifications carbamidomethylation (C) and oxidation (M) were chosen, DDA-MBR were performed with MS tolerance of 10 ppm and IM tolerance of 0.05 (1/k0).

The obtained data from mass spectrometry were processed using the standard Excel program. Cut-offs were applied at *p*-values less than 0.05 and at least a twofold change in expression. Proteins detected by mass spectrometry were only considered if at least one unique peptide was repeatedly found.

## Figures and Tables

**Table 1:** Assignment of selected proteins which are coordinately increased upon depletion of transketolase and transaldolase to specific cellular processes.

Functional Category/Protein	Protein Function	Ratio Tkl1 Depletion	Ratio Tal1 Depletion
**Peroxisomal proteins** **(fatty acid and triacylglycerol degradation)**			
Lpx1	triacylglycerol lipase of peroxisomes [19]	34.82	-
Faa2	fatty acyl-CoA synthetase [20]	19.81	43.11
Fox1 (Pox1)	fatty acyl-CoA oxidase [21]	7.07	13.41
Cta1	catalase A [22]	5.98	9.01
Fox2 (Pox2)	3-hydroxyacyl-CoA dehydrogenase/enoyl-CoA hydratase [23]	5.86	37.61
Mdh3	peroxisomal malate dehydrogenase [24]	2.35	4.39
Fox3 (Pot1)	3-ketoacyl-CoA thiolase [25]	-	13.41
**Carnitine shuttle**			
Crc1	carnitine transmembrane transporter [26]	13.75	21.46
Cat2	carnitine acetyl transferase (peroxisomes and mitochondria) [27]	8.17	24.89
Yat2	cytosolic carnitine acetyl transferase [28]	4.18	5.40
Yat1	carnitine acetyl transferase at the outer mitochondrial membrane [29]	3.16	3.16
**2-Methylcitrate pathway**			
Pdh1	2-methylcitrate dehydratase [30]	22.12	38.37
Icl2	2-methylisocitrate lyase [31]	9.21	21.77
Cit3	mitochondrial citrate synthase [32]	4.01	3.66
**Stress-induced proteins**			
Gac1	regulatory subunit of protein phosphatase [33]	-	64.00
Rny1	RNase; promotes apoptosis under stress [34]	44.22	-
Gre1	hydrophilin essential for rehydration [35]	-	31.27
Tma17	regulatory subunit of fatty acid synthase [36]	5.00	4.81
Srx1	sulfiredoxin, acts on thioredoxin peroxidase Tsa1 [37]	3.27	4.98
Gre3	aldose reductase involved in methylgyoxal metabolism [35]	3.01	2.46

Given are the ratios of the listed proteins from cells exposed to doxycycline versus those grown in the absence of the drug, from the strains, with growth conditions and bioinformatic parameters as described in the legend of Figure 5 and in Materials and Methods Section 4.6.

**Table 2:** Specific activities of transketolase and transaldolase.

Strain	Relevant Genotype	mU/mg Protein
**Transketolase**		
CBS2359	*TKL1*	269 ± 11
KBO23-2B	*tkl1::kanMX leu2::LEU2-TKL1*	241 ± 7
KBO22-4A	*tkl1::kanMX leu2::LEU2-ScTKL1*	136 ± 12
KBO24-9C	*tkl1::kanMX leu2::LEU2-ScTKL2*	b.d.
KBO25-2C	*tkl1::kanMX leu2::LEU2-ScPFK2p-HsTKL1*	75 ± 5
**Transaldolase**		
CBS2359	*TAL1*	149 ± 4
KBO28-5B	*tal1::kanMX leu2::LEU2-TAL1*	130 ± 5
KBO27-2A	*tal1::kanMX leu2::LEU2-ScTAL1*	37 ± 1
KBO30-2A	*tal1::kanMX leu2::LEU2-ScPFK2p-HsTAL1*	22 ± 2
Kl453-13A	*tal1::ScLEU2*	b.d.

Given are the mean specific activities and their standard deviations obtained from at least three biological replicates, with three technical replicates, each. Species designations are as follows: *Sc = Saccharomyces cerevisiae* and *Hs = Homo sapiens*, and genes from *Kluyveromyces lactis* are given without prefix. For complete genotypes and details on strain construction and enzyme assays, consult Materials and Methods. b.d. = below detection (<2 mU/mg protein).

**Table 3:** *Kluyveromyces lactis* strains used in this work.

Strain	Genotype
Diploid strains	
KHO70	*MATa*/*MATalpha ura3*/*ura3 leu2*/*leu2 his3::loxP*/*HIS3 ade2::loxP*/*ADE2 ku80::loxP*/*ku80::loxP*
KHO221	as KHO70 but with *tal1::ScLEU2*/*TAL1*
KHO344	*MATa*/*MATalpha ura3*/*URA3 leu2*/*LEU2 his3::loxP*/*his3::loxP rpe1::SpHIS5*/*RPE1 rho5::kanMX*/*RHO5*
KHO404	as KHO70 but with *gnd1::ScLEU2*/*GND1*
KHO410	as KHO70 but with *rki1::kanMX*/*RKI1*
KHO431	as KHO70 but with *shb17::ScLEU2*/*SHB17*
KHO489	*MATa*/*MATalpha ura3*/*URA3 leu2*/*leu2 fba1::kanMX*/*FBA1 shb17::ScLEU2*/*SHB17*
KHO494	*MATa*/*MATalpha ura3*/*URA3 leu2*/*LEU2 his3::loxP*/*his3::loxP zwf1::SpHIS5*/*ZWF1 ku80::loxP*/*KU80*
KHO507	*MATa*/*MATalpha ura3*/*URA3 leu2*/*LEU2 his3::loxP*/*his3::loxP sol4::SpHIS5*/*SOL4 ku80::loxP*/*KU80*
KHO526	*MATa/MATalpha ura3/ura3 leu2/leu2 his3::loxP/his3::loxP rki1::kanMX/RKI1 zwf1::SpHIS5/ZWF1 ku80::loxP/KU80 + pRKI1-URA3*
KHO528	*MATa*/*MATalpha ura3*/*ura3 leu2*/*LEU2 his3::loxP*/*his3::loxP gnd1::kanMX*/*GND1 zwf1::SpHIS5*/*ZWF1 ku80::loxP*/*KU80 + pGND1-URA3*
KHO554	*MATa*/*MATalpha ura3*/*URA3 leu2*/*leu2 leu2::LEU2-ScTAL1 tal1::kanMX*/*TAL1*
KHO555	*MATa*/*MATalpha ura3*/*URA3 leu2*/*leu2 leu2::LEU2-ScTKL1 tkl1::kanMX*/*TKL1 ku80::loxP*/*KU80*
KHO557	*MATa*/*MATalpha ura3*/*URA3 leu2*/*leu2 leu2::LEU2-TKL1 tkl1::kanMX*/*TKL1 ku80::loxP*/*KU80*
KHO559	*MATa*/*MATalpha ura3*/*URA3 leu2*/*leu2 leu2::LEU2-TAL1 tal1::kanMX*/*TAL1*
KHO561	as KHO70 but with *tkl1::ScLEU2*/*TKL1*
KHO574	*MATa*/*MATalpha ura3*/*URA3 leu2*/*leu2 leu2::LEU2-HsTAL1 tal1::kanMX*/*TAL1*
KHO577	*MATa*/*MATalpha ura3*/*URA3 leu2*/*leu2 leu2::LEU2-HsTKL1 tkl1::kanMX*/*TKL1 ku80::loxP*/*KU80*
KHO579	*MATa*/*MATalpha ura3*/*URA3 leu2*/*leu2 leu2::LEU2-ScTKL2 tkl1::kanMX*/*TKL1 ku80::loxP*/*KU80*
Kl1323	as KHO70 but with *tkl1::kanMX*/*TKL1*
Kl1324	as KHO70 but with *tal1::kanMX*/*TAL1*
Haploid strains	
CBS2369	Wild-type
KBO22-4A	*MATa ura3 his3::loxP leu2::LEU2-ScTKL1 tkl1::kanMX ku80::loxP*
KBO23-2B	*MATa ura3 leu2::LEU2-KlTKL1 tkl1::kanMX ku80::loxP*
KBO24-9C	*MATalpha his3::loxP leu2::LEU2-ScTKL2 tkl1::kanMX ku80::loxP*
KBO25-2C	*MATa ura3 his3::loxP ade2::loxP leu2::LEU2-ScPFK2-HsTKL1 tkl1::kanMX ku80::loxP*
KBO27-2A	*MATa ura3 leu2::LEU2-ScTAL1 Kltal1::kanMX*
KBO28-5B	*MATa his3::loxP leu2::LEU2-KlTAL1 Kltal1::kanMX*
KBO30-2A	*MATa leu2::LEU2-ScPFK2-HsTAL1 Kltal1::kanMX*
KHO69-8C	*MATalpha ura3 leu2 his3::loxP ku80::loxP*
KHO239-2C	*MATa ura3::tTA-SpHIS5 leu2 his3::loxP ade2::loxP lac4::loxP*
KHO382-1B	*MATalpha his3::loxP ura3::tTA-SpHIS5 kanMX-tetOFFp::KlTKL1*
KHO551-3B	*MATa his3::loxP ura3::tTA-SpHIS5 kanMX-tetOFF-KlTAL1*
Kl453-13A	*MATa ura3 leu2 tal1::ScLEU2 ku80::loxP*

All strains employed derive from the type strain CBS2359 obtained in a congenic series as described in [13]. Strains carrying a *ku80* deletion were employed to facilitate homologous recombination for genomic manipulations. The deletion allele was eliminated by backcrosses to wild-type strains, if not stated otherwise.

## Data Availability

Protein and nucleic acid sequences are available from the public databases as cited in [6]. Sequences of plasmids constructed and manipulated chromosomal loci are available upon request.

## Supplementary Materials

The following supporting information can be downloaded at: https://www.mdpi.com/article/10.3390/ijms26030938/s1.

## Abbreviations

5-FOA = 5-fluoro orotic acid; PCR = polymerase chain reaction; PPP = pentose phosphate pathway; and ZWF = gene encoding G6PDH (“Zwischenferment”).
